# Deoxynivalenol and Its Modified Forms: Are There Major Differences?

**DOI:** 10.3390/toxins8110334

**Published:** 2016-11-16

**Authors:** Arash Alizadeh, Saskia Braber, Peyman Akbari, Aletta Kraneveld, Johan Garssen, Johanna Fink-Gremmels

**Affiliations:** 1 Division of Veterinary Pharmacology, Pharmacotherapy and Toxicology, Institute for Risk Assessment Sciences, Utrecht University, Yalelaan 104, 3584-CM Utrecht, The Netherlands; a.alizadeh@uu.nl (A.A.); a.peyman@uu.nl (P.A.); a.d.kraneveld@uu.nl (A.K.); j.fink@uu.nl (J.F.-G.); 2 Division of Pharmacology, Utrecht Institute for Pharmaceutical Sciences, Faculty of Science, Utrecht University, Universiteitsweg 99, 3584-CG Utrecht, The Netherlands; j.garssen@uu.nl; 3 Department Immunology, Nutricia Research, Uppsalalaan 12, 3584-CT Utrecht, The Netherlands

**Keywords:** mycotoxin, deoxynivalenol, 3-acetyl-deoxynivalenol, 15-acetyl-deoxynivalenol, de-epoxy-DON, DON-3-*O*-glucoside, intestinal barrier, CXCL8

## Abstract

Considering the diverse toxic effects of the *Fusarium* toxin deoxynivalenol (DON), its common occurrence in wheat-based products, and its stability during processing, DON constitutes an increasing health concern for humans and animals. In addition to the parent compound DON, human and animal exposure encompasses the acetylated fungal metabolites 3-acetyl-deoxynivalenol (3ADON) and 15-acetyl-deoxynivalenol (15ADON) as well as the plant-derived DON-glucoside (DON3G) and the bacterial product de-epoxy-DON (DOM-1). In the current study we used the well-established Caco-2 cell model to compare the effects of these naturally occurring forms of DON on cell viability and markers of barrier integrity, as well as on the release of the pro-inflammatory chemokine chemokine CXC motif ligand (CXCL8). Results show that 3ADON is less potent in inducing adverse effects on barrier integrity when compared to DON, whereas 15ADON appears to be slightly more potent than DON. In contrast, DON3G and DOM-1 exerted no measurable adverse effects on the intestinal barrier. It was also demonstrated that galacto-oligosaccharides (GOS) are able to protect epithelial cells against DON and its acetylated forms, which suggests that GOS are beneficial food additives in the protection of vulnerable segments of the human population against adverse effects of DON and its derivatives.

## 1. Introduction

**Figure 1:**
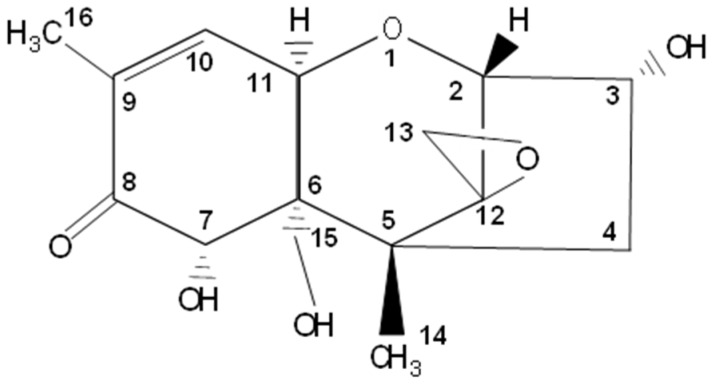
Deoxynivalenol (DON).

Trichothecenes comprise a large family of structurally related mycotoxins produced by various *Fusarium* species. Chemically, trichothecenes belong to the group of sesquiterpenoids and share a 9, 10 double bond and the 12, 13 epoxide group, the latter being considered to be critical for their toxicity [1,2,3] (Figure 1). The mycotoxin deoxynivalenol (DON), mainly produced by *Fusarium graminearum* and *Fusarium culmorum*, is one of the main representatives of the type B trichothecenes and is currently among the most prevalent and important contaminants of cereals and cereal-based products [4,5,6]. Recently initiated monitoring of urine samples for the presence of DON confirms the high rate of exposure, albeit in many cases at low levels [5,7,8].

The first target of DON toxicity seems to be the intestinal tract, as DON is able to impair the intestinal barrier by affecting the expression and assembly of tight junctions and initiating an inflammatory response [9]. Subsequently, DON can increase the paracellular transport of luminal antigens and even pathogens in vitro and in vivo [10,11,12], and in humans DON may even stimulate the development of allergies, including whey allergy as demonstrated in a murine model [13].

Fusaria produce not only DON, but also two prominent acetylated derivatives of DON, 3-acetyl-deoxynivalenol (3ADON) and 15-acetyl-deoxynivalenol (15ADON) [5,14,15,16]. Nowadays *F. graminearum* genotypes producing all three forms (DON, 3ADON and 15ADON) have been identified [17]. Plants in turn aim to protect their tissues, conjugating free DON to one or more molecules of glucose of which DON-3-β-d-glucoside (DON3G) is the most frequently measured conjugate [14,18,19]. The glycosylation changes the physico-chemical properties of DON significantly, and hence such plant glucosides have long remained undetected. They have been described as “masked” mycotoxins, as they escape common extraction and detection methods validated for the determination of DON [18,19,20]. The concentrations of DON3G in wheat and maize samples are highly variable, and Berthiller et al. [20,21] reported in some individual studies that DON3G concentrations even exceeded those of DON. Upon ingestion with food, the glucoside binding can be cleaved readily by bacterial glucosidases. Gratz et al. [22] reported that mixed human fecal microbiota are capable of hydrolyzing 80% of DON3G in 4 h. Comparable data for other DON glucosides are not available, but it is generally assumed that liberated DON contributes to overall human and animal exposure [14,18,19,23].

The intestinal microbiome does not only liberate DON from plant material, but is also able to convert DON to its de-epoxide, denoted DOM-1 [3,24]. Although earlier studies failed to detect significant amounts of DOM-1 in human blood samples, DOM-glucuronides have been detected in human urine samples and recently in urine samples of children as well. These findings indicate that part of the formed DOM-1 is absorbed and subsequently excreted via the kidneys [7]. All the above mentioned derivatives and metabolites of DON are summarized in Table 1.

The aim of the current experiments was a direct comparison of the different DON-derivatives in a standardized Caco-2 cell model that is widely accepted in order to study direct effects of drugs and toxins on the epithelial barrier. In parallel, we tested the protective effect of a defined formulation of non-digestible oligosaccharides under the same experimental conditions. These galacto-oligosaccharides (GOS) are produced from lactose and are widely used in infant diets due to their prebiotic and immuno-modulating properties [25,26,27,28,29,30]. In previous in vitro and in vivo experiments we have already demonstrated that GOS are able to protect the breakdown of the intestinal barrier following exposure to DON [28]. This protective effect of GOS concerns not only the maintenance of barrier integrity measured by trans-epithelial electrical resistance (TEER) and paracellular transport of marker molecules, such as Lucifer yellow (LY), but also an anti-inflammatory effect, preventing the DON-induced increase in the secretion of the pro-inflammatory chemokine chemokine CXC motif ligand (CXCL8).

## 2. Results

### 2.1. Comparison of Lactate Dehydrogenase (LDH) Leakage Induced by DON and Its Derivatives and Metabolites

**Figure 2:**
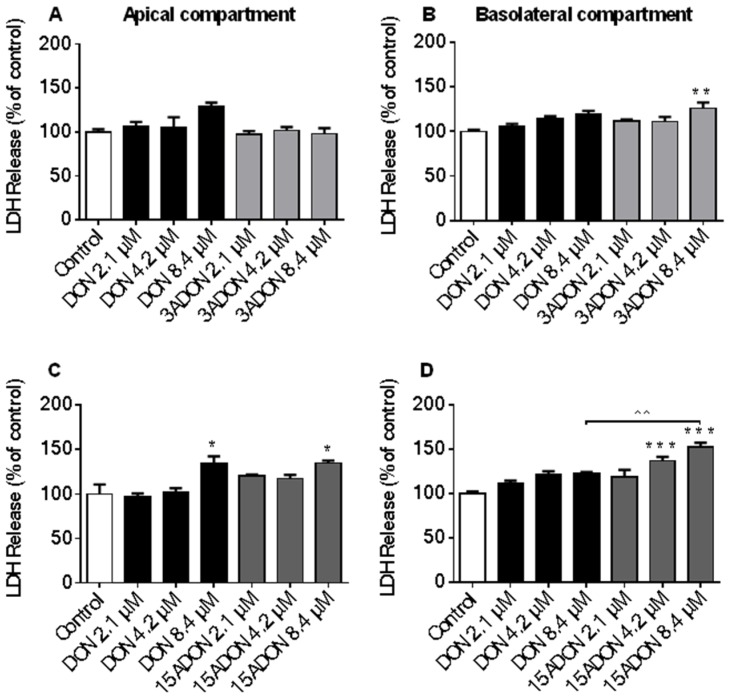
Cytotoxic effects of DON, and its acetylated derivatives in Caco-2 cells. Differentiated Caco-2 cells on transwell inserts were exposed from the apical and basolateral compartment to increasing DON, 3-acetyl-deoxynivalenol (3ADON) (**A**,B****) and 15-acetyl-deoxynivalenol (15ADON) (**C**,**D**) concentrations (2.1, 4.2, 8.4 µM) for 24 h, followed by evaluation of lactate dehydrogenase (LDH) release into the apical (**A**,**C**) and basolateral (**B**,**D**) compartment. Results are expressed as a percentage of LDH released by the control group as mean ± standard error of the mean (SEM). * *p* < 0.05, ** *p* < 0.01, *** *p* < 0.001; significantly different from control group; ^^ *p* < 0.01; significantly different from corresponding DON group.

**Figure 3:**
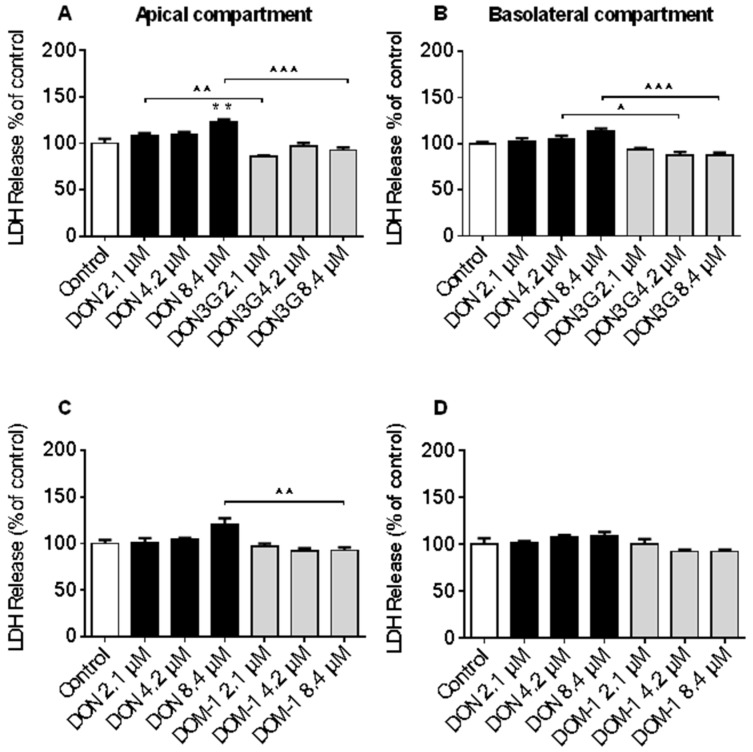
Cytotoxic effects of DON, DON3G and DOM-1 in Caco-2 cells. Differentiated Caco-2 cells on transwell inserts were exposed from the apical and basolateral compartment to increasing DON, DON-3-*O*-glucoside (DON3G) (**A**,B****) and de-epoxy-DON (DOM-1) (**C**,**D**) concentrations (2.1, 4.2, 8.4 µM) for 24 h, followed by evaluation of LDH release into the apical (**A**,**C**) and basolateral (**B**,**D**) compartment. Results are expressed as a percentage of LDH released by the control group as mean ± SEM. ** *p* < 0.01; significantly different from control group; ^ *p* < 0.05, ^^ *p* < 0.01, ^^^ *p* < 0.001 significantly different from corresponding DON group.

Non-specific cytotoxicity was determined by measuring the LDH leakage from differentiated Caco-2 cells. Results are presented individually for the apical and basolateral compartment. LDH leakage measured at the apical compartment following exposure to 8.4 µM DON or 15ADON was comparable, while no effect of 3ADON on LDH release into the apical compartment was observed in comparison to control (Figure 2A,C). In contrast, 3ADON (8.4 µM) and 15ADON (4.2 and 8.4 µM) significantly increased LDH release into the basolateral compartment (Figure 2B,D), whereas DON did not significantly affect LDH release into the basolateral compartment. Comparable experiments with DON3G and DOM-1 using the same concentration range did not result in any significant changes in LDH leakage compared to control (Figure 3A–D). Even at a higher concentration (16.8 µM), DON3G did not significantly affect LDH leakage (Figure S1). However, 16.8 µM DOM-1 increased LDH release in both compartments and these effects were not significantly different from the control group.

### 2.2. Comparison of Changes in Trans-Epithelial Resistance Induced by DON and Its Derivatives and Metabolites

**Figure 4:**
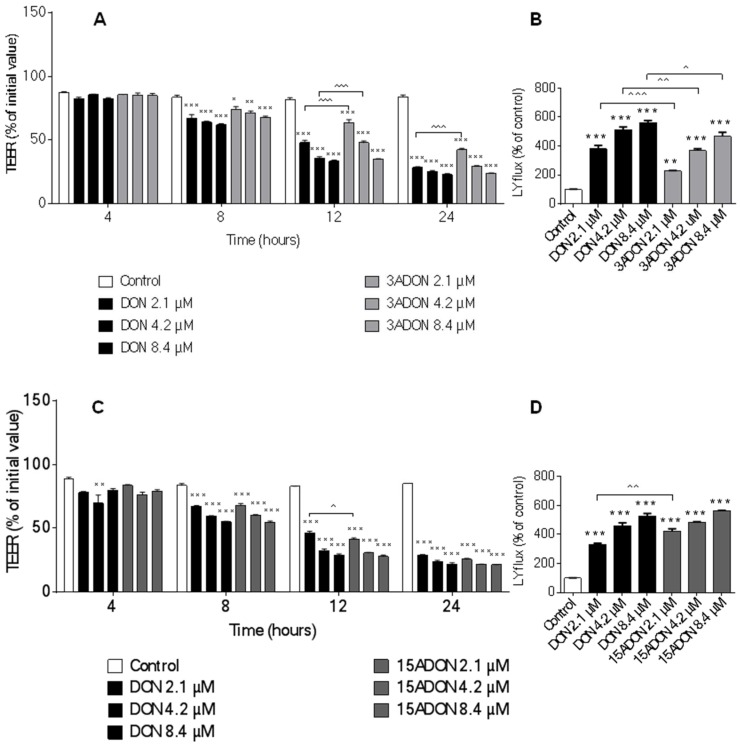
Effects of DON and its acetylated derivatives on trans-epithelial electrical resistance (TEER) and Lucifer yellow (LY) translocation. Differentiated Caco-2 cells on transwell inserts were exposed from the apical and basolateral compartment to increasing concentrations of DON, 3ADON (**A**,B****) and 15ADON (**C**,**D**) for 24 h and TEER values were measured during different time points (**A**,**C**). Subsequently, LY translocation from the apical to the basolateral compartment was measured (**B**,**D**). Results are expressed as percentage of initial value (TEER values) or percentage of control group (LY translocation) as mean ± SEM. * *p* < 0.05, ** *p* < 0.01, *** *p* < 0.001; significantly different from corresponding control group, ^ *p* < 0.05, ^^ *p* < 0.01, ^^^ *p* < 0.001; significantly different from corresponding DON group.

TEER is a commonly used marker for the integrity of the tight cellular monolayer. In the current experiments, 3ADON and 15ADON concentration-dependently decreased the TEER values in a manner comparable to DON effects at different time points (Figure 4A,C). However, 3ADON is slightly less potent in inducing a decrease in TEER values compared to DON, whereas 2.1 µM 15ADON appears to be even more potent in decreasing TEER values compared to 2.1 µM after 12 h of exposure.

**Figure 5:**
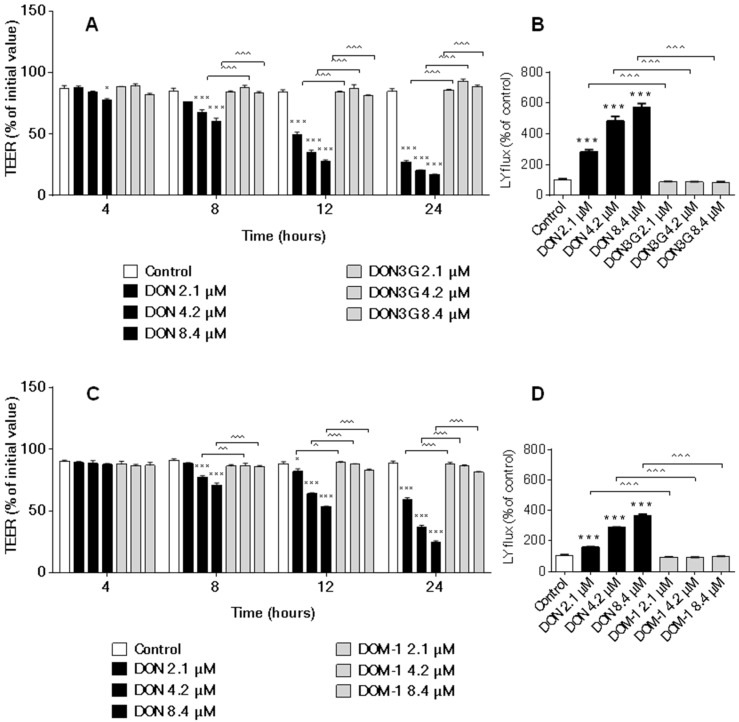
Effects of DON, DON3G and DOM-1 on TEER and LY translocation. Differentiated Caco-2 cells on transwell inserts were exposed from the apical and basolateral compartment to DON, DON3G (**A**,B****) and DOM-1 (**C**,**D**) for 24 h and TEER values were measured during different time points (**A**,**C**). Subsequently, LY translocation from the apical compartment to the basolateral compartment was measured (**B**,**D**). Results are expressed as percentage of initial value (TEER values) or percentage of control group (LY translocation) as mean ± SEM. * *p* < 0.05, *** *p* < 0.001; significantly different from corresponding control group, ^ *p* < 0.05, ^^ *p* < 0.01, ^^^ *p* < 0.001 significantly different from corresponding DON group.

In the second series of experiments, a comparison between the effects of DON and DON3G or DOM-1 was made and neither of the modified forms showed a significant effect on TEER in the tested concentration range (Figure 5A,C). However, there was a slight decrease in TEER values after exposure to 8.4 µM DOM-1 for 12 and 24 h.

### 2.3. Comparison of Changes in Paracellular Transport across the Monolayer Induced by DON and Its Derivatives and Metabolites

The paracellular transport of Lucifer yellow across the Caco-2 monolayer was measured as a functional parameter of an impaired barrier function. As expected on the basis of the TEER measurements, 3ADON and 15ADON concentration-dependently increased Lucifer yellow permeability in a manner comparable to that of DON (Figure 4B,D). Minor differences were observed as 3ADON was less effective in increasing the Caco-2 monolayer permeability compared to DON, whereas 2.1 µM 15ADON had a more pronounced effect on Lucifer yellow translocation than DON (Figure 4B,D). Exposure of cells to DON3G and DOM-1 did not result in measurable changes of Lucifer yellow transport from the apical to basolateral compartment (Figure 5B,D).

### 2.4. Comparison of Changes in CXCL8 Secretion Induced by DON and Its Derivatives and Metabolites

**Figure 6:**
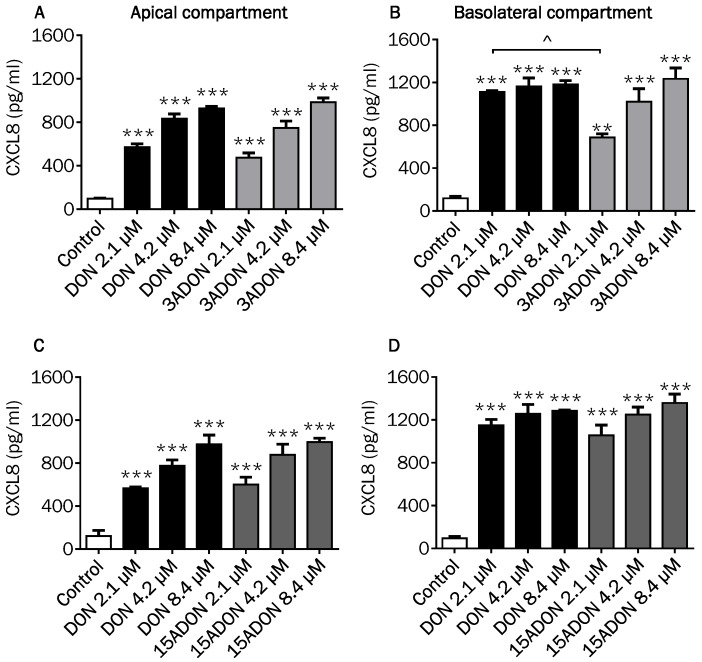
Effects of DON, and its acetylated derivatives on chemokine CXC motif ligand (CXCL8) secretion. Differentiated Caco-2 cells on transwell inserts were exposed from the apical and basolateral compartment to DON, 3ADON (**A**,B****) and 15ADON (**C**,**D**) for 24 h, followed by evaluation of CXCL8 secretion into the apical (**A**,**C**) and basolateral (**B**,**D**) compartment. Results are expressed as pg/mL as mean ± SEM. ** *p* < 0.01, *** *p* < 0.001; significantly different from control group; ^ *p* < 0.05 significantly different from corresponding DON group.

**Figure 7:**
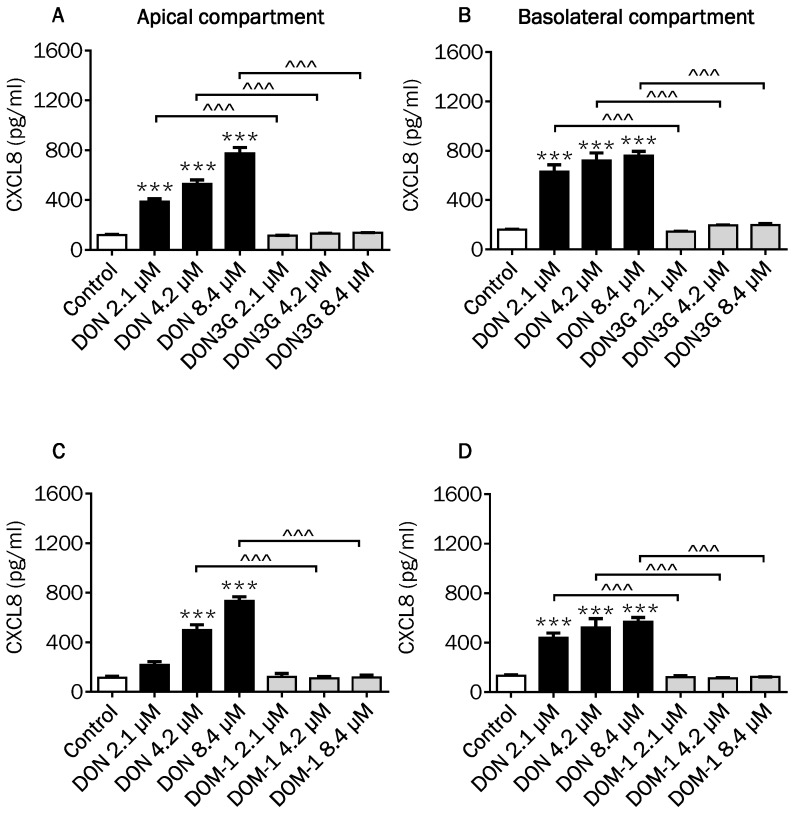
Effects of DON, DON3G and DOM-1 on CXCL8 secretion. Differentiated Caco-2 cells on transwell inserts were exposed from the apical and basolateral compartment to DON, DON3G (**A**,B****) and DOM-1 (**C**,**D**) for 24 h, followed by evaluation of CXCL8 secretion into the apical (**A**,**C**) and basolateral (**B**,**D**) compartment. Results are expressed as pg/mL as mean ± SEM. *** *p* < 0.001; significantly different from control group; ^^^ *p* < 0.001 significantly different from corresponding DON group.

DON as well as 3- and 15ADON concentration-dependently induced the secretion of the pro-inflammatory chemokine CXCL8 in the apical compartment of the Caco-2 cell model (Figure 6A,C). In the basolateral compartment, the secretion of CXCL8 was also increased by DON, while a dose-dependent increase was observed by 3ADON and 15ADON (Figure 6B,D). No further remarkable differences were observed between the individual DON forms except that 2.1 µM 3ADON was less potent in stimulating the CXCL8 release into the basolateral compartment compared to 2.1 µM DON (Figure 6B). Comparable experiments were conducted with DON3G and DOM-1, again without any significant response on the CXCL8 secretion, confirming that these forms of DON are much less active than the parent DON and its acetylated forms (Figure 7A–D).

### 2.5. GOS Prevent and Suppress Barrier Disruption and Pro-Inflammatory Effects Exerted by DON and Its Acetylated Derivatives

**Figure 8:**
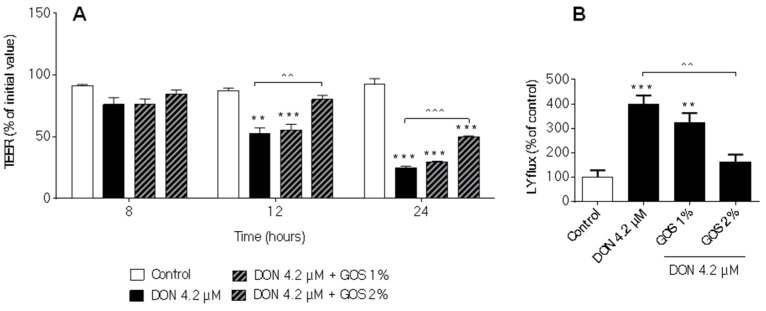

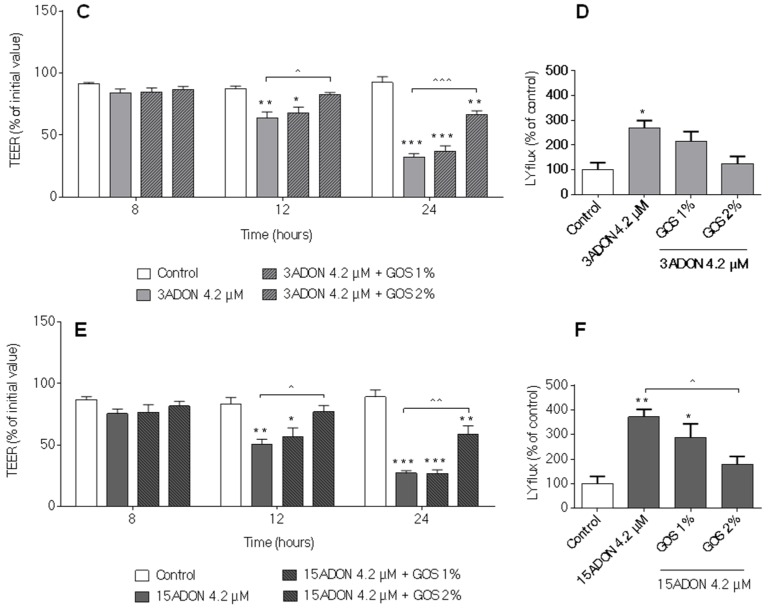
Effects of galacto-oligosaccharides (GOS) on the disruption of intestinal barrier induced by DON and its acetylated derivatives. Differentiated Caco-2 cells on transwell inserts were pretreated from the apical and basolateral compartment with GOS (1% and 2%) for 24 h, followed by DON (**A**,**B**); 3ADON (**C**,**D**) and 15ADON (**E**,**F**) exposure for 24 h at a concentration of 4.2 µM and TEER values were measured during different time points (**A**,**C**,**E**). Subsequently, the LY translocation from the apical to the basolateral compartment was measured (**B**,**D**,**F**). Results are expressed as percentage of initial value (TEER values) or percentage of control group (LY translocation) as mean ± SEM. * *p* < 0.05, ** *p* < 0.01, *** *p* < 0.001; significantly different from corresponding control group. ^ *p* < 0.05, ^^ *p* < 0.01, ^^^ *p* < 0.001; significantly different from corresponding DON, 3ADON and 15ADON group.

**Figure 9:**
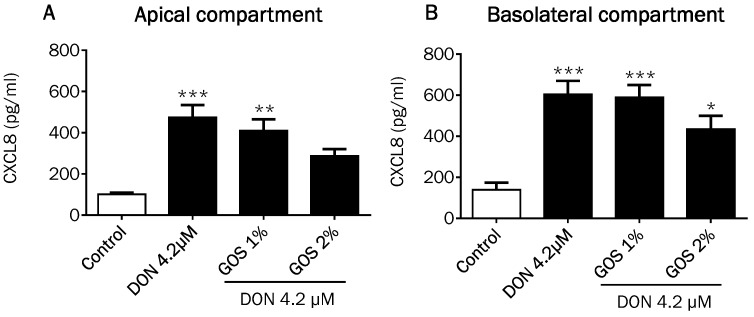

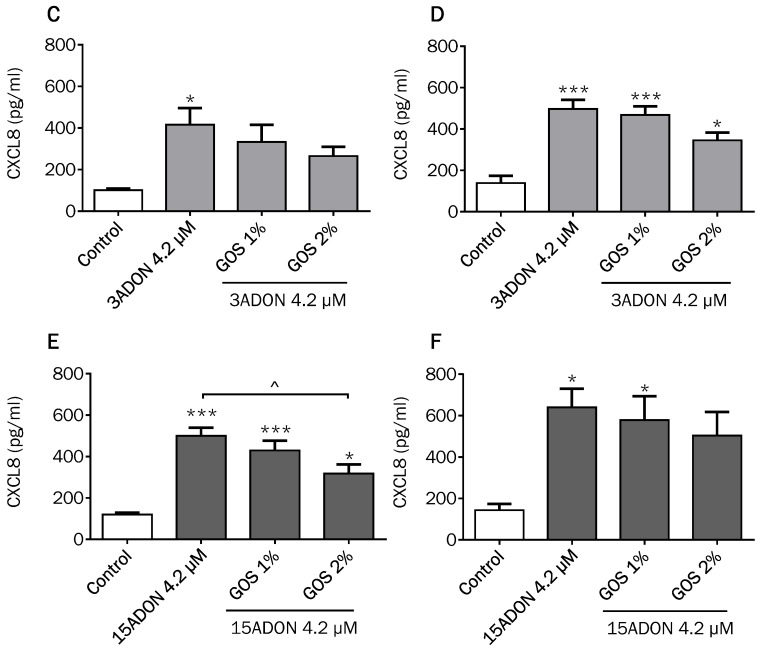
Effects of GOS on the CXCL8 release induced by DON and its acetylated derivatives. Differentiated Caco-2 cells on transwell inserts were pretreated from the apical and basolateral compartment with GOS (1% and 2%) for 24 h, followed by DON (**A**,B****), 3ADON (**C**,D****) and 15ADON (**E**,F****) exposure for 24 h at a concentration of 4.2 µM and secretion of CXCL8 into the apical (**A**,**C**,**E**) and basolateral (**B**,**D**,**F**) compartment was measured. Results are expressed as pg/mL as means ± SEM. * *p* < 0.05, ** *p* < 0.01, *** *p* < 0.001; significantly different from control group; ^ *p* < 0.05 significantly di**f**ferent from 15ADON group.

In consideration of our previous results, we finally wanted to confirm that GOS are also able to suppress effects of DON and its biologically active acetylated forms. In Figure 8 and Figure 9, composite panels summarizing the results obtained with TEER measurement, Lucifer yellow permeability and CXCL8 secretion are presented. Compared to the effects of GOS on the DON-induced TEER decrease and LY increase (Figure 8A,B), 2% GOS were also able to attenuate the 3ADON and 15ADON-induced TEER decrease at 12 h and 24 h and the corresponding Lucifer yellow flux (Figure 8C–F), whereas 1% GOS did not show significant preventive effects against DON and its acetylated derivatives. The GOS preventive effects in 3ADON- and 15ADON-stimulated cells after 24 h stimulation were more pronounced compared to 12-h stimulation (Figure 8C,E). The main finding of these experiments is that 2% GOS was equally effective in the suppression of 3A- and 15ADON effects as compared to DON. Comparable results were observed for the CXCL8 release (Figure 9).

## 3. Discussion

Considering the diverse toxic effects of the trichothecene mycotoxin DON and its common occurrence in small grains—in particular wheat—as well as its stability during food and feed processing, DON constitutes an increasing health concern for humans and animals [15,31,32]. In addition to the parent DON molecule, humans and animals may be exposed to additional fungal derivatives of DON, such as 3ADON and 15ADON. Next to these fungal derivatives, human and animals are also exposed to plant derived DON-glucosides, including DON3G. These conjugates are expected to have a low toxicity, but may contribute to overall exposure [5,19,21,23].

The first aim of the current study was to compare the different modified forms of DON in a well-established Caco-2 cell model. The selected endpoints of toxicity were barrier function measured by TEER and paracellular transport of the marker Lucifer yellow, as well as the DON-induced inflammatory response, measured as CXCL8 release.

The cytotoxicity of DON derivatives and metabolites was measured by means of LDH leakage following exposure of Caco-2 cell monolayers from the basolateral as well as the apical side. These cytotoxicity results demonstrated that DON and its acetylated derivatives 3ADON and 15ADON induced no immediate cytotoxicity up to a concentration of 4.2 µM. Akbari et al. [10] also reported that Caco-2 cells are resilient to DON in a concentration range up to 12.5 µM following exposure for 24 h. Previous studies demonstrated no significant effects on Caco-2 cell viability in a concentration range of 0.337 to 33.7 µM DON (48 h) and 0.337 to 33.7 µM DON acetylated derivatives (6 h) [16,33]. For 3ADON a significant increase in extracellular LDH was measured at the highest concentration (8.4 µM) tested, whereas for 15ADON significantly increased values, exceeding even those measured for DON, were observed at 4.2 and 8.4 µM. The latter finding might indicate a more rapid cellular uptake of the acetylated derivatives.

In contrast, neither DON3G nor DOM-1 exerted any LDH leakage over the entire concentration range up to the highest concentration of 8.4 µM. In addition, even a higher concentration (16.8 μM) of DON3G and DOM-1 did not significantly impair cell viability, however, there was a trend where 16.8 µM DOM-1 increased the LDH leakage. These data suggest that the bacterial metabolite DOM-1 could be cytotoxic for intestinal epithelial cells at high concentrations. Furthermore, recently published data observed no cytotoxic effects of DON3G (0.05 µM to 10 µM) after 48 h exposure in proliferating Caco-2 cells, whereas differentiated Caco-2 cells showed no cytotoxicity up to 100 µM for 8 days [34].

The decrease in TEER values and increase in Lucifer yellow permeability as assessed in the current study provide strong evidence that DON and its acetylated derivatives (3ADON and 15ADON) have the potency to alter the permeability of Caco-2 cells. Reduction in TEER values following exposure to DON have been reported in several studies with different cell lines, including Caco-2 [10,11,12,35] and two different intestinal porcine columnar epithelial cell lines (IPEC-1 [12] and IPEC-J2 [36]), but studies with DON derivatives are scarce. It has been reported that 15ADON is more potent in affecting TEER values in IPEC-1 [37] and in Caco-2 cells [16] compared to DON and 3ADON. In contrary to these studies, the current results demonstrated that DON and 15ADON induce similar effects on TEER, while at a concentration of 2.1 µM the effect of 15ADON on the TEER exceeded that of DON (after 12 h exposure). The adverse effects on the epithelial integrity of 3ADON were less pronounced compared to DON.

These differences between the individual studies might be due to the different cell lines (IPEC-1 vs. Caco-2 cells), exposure route (apical exposure vs. exposure from both sides in our study) as well as the duration of exposure [16,37].

As hypothesized, DON3G had no effect on TEER values, which is line with previous results in which Caco-2 cells were exposed to 10 µM DON3G for 8 days [34]. Although there was a slight decrease in TEER after exposure to 8.4 µM DOM-1 for 12 and 24 h, DOM-1 did not significantly influence TEER values over the entire concentration range tested.

The TEER results of the current study are compatible with the Lucifer yellow permeability data. Increasing concentrations of DON, 15ADON and 3ADON resulted in enhanced paracellular translocation of the marker Lucifer yellow from the apical to the basolateral site, and a direct comparison revealed that the effect of 3ADON was less pronounced than that of DON, whereas 15ADON closely resembled the effects of DON.

Again, as expected DON3G and DOM-1, which did not affect TEER values, did also not result in an increase in the paracellular transport of Lucifer yellow.

As CXCL8 is pivotal for the progress of most local intestinal inflammatory reactions [38], the CXCL8 release into the supernatant of Caco-2 cells exposed to DON and its derivatives and metabolites was examined. The results resemble to a large extent the results of the markers of barrier integrity. A highly significant increase in CXCL8 was measured for DON and both acetylated forms. The CXCL8 secretion at the basolateral compartment induced by the different DON concentrations might reflect the biologically maximum CXCL8 response of the cells, as no clear concentration-dependency could be observed. Our observations of the DON-induced CXCL8 release are in agreement with previous studies, where DON had the potency to induce CXCL8 secretion by Caco-2 cells [11,28,39].

The results of the comparable experiment with DON3G and DOM-1 confirmed that DON3G had no effect on CXCL8 secretion, which was previously reported by measuring the CXCL8 mRNA expression [34].

Taken together, our current results confirm that the DON3G has no significant biological activity on the tested functional parameters of the intestinal barrier. Recent elegant in silico and in vitro experiments indicated that DON3G is apparently poorly absorbed by intestinal cells and fails to bind to the A-site of the ribosome peptidyl transferase center, and hence is not able to induce the activation of mitogen activated protein kinases (MAPK), which is the main pathway resulting in the typical ribotoxic stress considered as main molecular mechanism of DON-induced cell injury and inflammation [34].

From numerous other plant secondary metabolites that are present as glucosides, it is known that the oral bioavailability is low, which also applies to DON3G [40]. However, when these glucosides reach the lower parts of the intestine with a higher microbiota density, the glucosides are cleaved to a large extend by bacterial glucosidases, which leads to the release of the parent DON molecule.

The liberated DON contributes to human and animal exposure particularly in the large intestine, from which it also can be absorbed [34]. The actual concentration of free DON that reaches via this secondary route to the systemic circulation is expected to be highly variable, as bacterial de-epoxidases can rapidly convert the released DON to DOM-1, which is significantly less toxic [41]. DOM-1 has also been detected in human urine samples [42,43].

Comparing DON and its acetylated forms, it has been suggested that acetylated DON-forms are more rapidly absorbed from the intestines [16], a process that would be initiated by a more rapid uptake by intestinal cells. At the cellular level, DON inhibits protein synthesis via triggering a ribotoxic stress response through its high binding affinity to the peptidyl transferase region of the ribosome leading to the activation of the MAPK as mentioned above [9,44]. Pinton et al. [37] showed differences in toxicity between DON, 3ADON and 15ADON related to their ability to activate the MAPK and concluded that 15ADON is a more potent inducer of MAPKs. Comparable effects were also confirmed in vivo, where 15ADON induced more pronounced changes in the intestinal architecture [37].

In recent approaches towards the establishment of health-based guidance levels, an integral approach for the toxicological assessment of the parent mycotoxin and its modified forms (the general term for all identified metabolites) has been applied [45]. Such an approach was recently published by the European Food Safety Authority (EFSA) regarding the mycotoxin zearalenone and its modified forms, resulting in a so-called group health based guidance level [46]. Applying this approach to DON, the current results suggest that a toxic equivalent factor of >1 should be used for 15ADON co-occurring with DON in food materials, whereas the equivalent factor for 3ADON should be slightly <1. However, for the ultimate determination of the toxic equivalent factors in risk assessment for 3ADON and 15ADON, all other existing studies and data need to be taken into account.

For DON3G and DOM-1, toxic equivalent factors would not be necessary (or close to zero), but the microbiota-dependent cleavage of DON3G needs to be considered in the overall exposure assessment.

The second objective of this study was to investigate whether or not the already demonstrated protective effect of GOS on the DON-induced impairment of intestinal barrier function could also be expected for other biologically active forms of DON, such as 3ADON and 15ADON. The protective effects of GOS on DON-induced breakdown of barrier integrity have been previously demonstrated by our group in the same Caco-2 cell model [28]. The obtained results confirm this protective effect of 2% GOS not only against DON, but also against 3ADON and 15ADON, whereas the lower concentration of 1% GOS showed no significant preventive effects.

Recent epidemiological data suggest an extremely high frequency of exposure, but concentrations in human food still remain low and within the statutory limits. However, even at such low food concentrations, the effects of DON on intestinal barrier integrity remain of concern, as this might result in chronic inflammatory disease, including inflammatory bowel disease in humans, and in an increased allergic response to certain food allergens. This is of particular importance, as our group already demonstrated that DON increased the risk of whey allergy [13].

## 4. Materials and Methods

### 4.1. Caco-2 Culture

The human intestinal Caco-2 cell line was obtained from American Type Tissue Collection (Code HTB-37) (passages 5–19; Manassas, VA, USA). The cells were cultured in 75-cm^2^ culture flasks (Greiner Bio-One, Frickenhausen, Germany). Dulbecco’s modified Eagle’s minimum essential medium (DMEM), supplemented with 25 mM 4-(2-hydroxyethyl)-1-piperazineëthanesulfonic acid (HEPES), 4.5 g/L glucose (Gibco, Invitrogen, Carlsbad, CA, USA), 10% (*v*/*v*) inactivated fetal calf serum (FCS) (Gibco), glutamine (2 mM, Biocambrex, Verviers, Belgium), 1% (*v*/*v*) nonessential amino acids (Gibco) and penicillin (100 U/mL)/streptomycin (100 µg/mL) was utilized as medium for the growing cells. The cells were preserved in an incubator to provide them with the optimum moisture and temperature (humidified atmosphere of 95% air and 5% CO_2_ at 37 °C). The Caco-2 cells were grown on high-pore-density polyethylene terephthalate membrane transwell inserts (BD Biosciences, Franklin Lakes, NJ, USA) placed in a 24-well plate according to the protocol described by Akbari et al. [10].

### 4.2. Mycotoxins

DON, 15ADON, 3ADON and DOM-1 (Sigma-Aldrich, St Louis, MO, USA) and DON3G (Romer Labs GmbH, Tulln, Austria) were diluted in absolute ethanol (99.9%; JT Baker) to prepare the stock solution. Working dilutions were prepared in cell culture medium.

### 4.3. GOS

The commercially available Vivinal^®^ GOS syrup (FrieslandCampina Domo, Borculo, The Netherlands), comprising 45% GOS with a DP (degree of polymerization) of 2–8, 16% free lactose, 14% glucose, and 25% water, was used. Dilutions of GOS (1% and 2%) were prepared in complete cell culture medium. Previous experiments confirmed that GOS did not induce any cytotoxicity in Caco-2 cells at the selected concentrations [28].

### 4.4. Co-Exposure Experiments with Mycotoxins and GOS

The Caco-2 cells were grown on transwell culture inserts and exposed from the apical and basolateral side to different concentrations of DON, 15ADON, 3ADON, DON3G and DOM-1 for 24 h in a concentration range of 2.1 to 8.4 µM.

Additionally, in parallel experiments, Caco-2 cells were pretreated with GOS (1% and 2%) for 24 h (added to both the apical and basolateral compartments) and then challenged by either 4.2 µM DON or 4.2 µM DON fungal derivatives (3ADON and 15ADON) from apical and basolateral side in the presence of GOS for the next 24 h according to the protocol described in our previous studies [10,28].

### 4.5. LDH Assay

Caco-2 cells were exposed to DON, 3ADON, 15ADON, DON3G and DOM-1 (2.1, 4.2 and 8.4 µM) for 24 h and the cytotoxicity of the mycotoxins was evaluated by measuring the LDH release in the supernatant of apical and basolateral compartments using the CytoTox 96 non-radioactive cytotoxicity assay kit (Promega Corporation, Madison, WI, USA) according to the manufacturer’s instructions.

### 4.6. TEER Measurement

The Caco-2 monolayer integrity was assessed by TEER measurement utilizing Millicell-ERS volt-ohm meter (Millipore) at different time points (4, 8, 12 and 24 h) by using increasing concentrations of DON, 3ADON, 15ADON, DON3G and DOM-1 (2.1 to 8.4 µM). In line with these experiments the cells were pre-incubated with GOS (1% and 2%) for 24 h followed by DON, 3ADON and 15ADON challenge (4.2 µM) for 24 h and TEER values were recorded at different time points (8 h, 12 h and 24 h).

### 4.7. Paracellular Permeability Assay

For the determination of the paracellular permeability, the flux of Lucifer yellow (LY, molecular mass of 0.457 kDa, Sigma Chemical Co., St. Luis, MO, USA) was evaluated. The transport studies were conducted by adding 16 µg/mL of Lucifer yellow to the apical compartment (350 µL) of the transwell inserts for 4 h and measuring the paracellular flux to the basolateral compartment by quantifying the fluorescence intensity using a fluorometer (FLUOstar Optima, BMG Labtech, Offenburg, Germany) at excitation and emission wavelengths of 410 and 520 nm, respectively.

### 4.8. CXCL8

The amount of CXCL8 release by Caco-2 cells into the apical and basolateral compartments of the transwell inserts was quantitatively determined by the human IL-8 ELISA Set (BD Biosciences, San Diego, CA, USA) in accordance with the manufacturer’s instructions.

### 4.9. Statistical Analysis

The results from all mycotoxin comparison studies were expressed as mean ± standard error of the mean (SEM) of three independent wells (three wells/condition), whereas the results of all mycotoxin studies in combination with GOS were expressed as mean ± SEM of four independent experiments (*n* = 4), each performed in triplicate (three wells/condition). Differences between results were statistically evaluated using One-way ANOVA with Bonferroni post-hoc test.

## Figures and Tables

**Table 1:** Deoxynivalenol (DON) and its common modified forms.

Common DON Derivatives and Metabolites	Origin	Structure
DON	Fungi	(3α,7α)-3,7,15-trihydroxy-12,13-epoxy-trichothec-9-en-8-one
3-acetyl-DON (3ADON)	Fungi	-C_2_H_3_O (Acetyl)
15-acetyl-DON (15ADON)	Fungi	-C_2_H_3_O (Acetyl)
De-epoxy-DON (DOM-1)	Bacterial metabolite of DON	-CH_2_ (at C12)
DON-3-β-d-glucoside (DON3G)	Plant conjugates of DON	-C_6_H_11_O_5_ (Glucoside)

## Supplementary Materials

The following are available online at www.mdpi.com/2072-6651/8/11/334/s1, Figure S1: Cytotoxic effects of DON, DON3G and DOM-1 in Caco-2 cells.
