# Supplementary material for: Deoxynivalenol and Its Modified Forms: Are There Major Differences?
Source: Toxins (Basel). 2016 Nov 16;8(11):334. doi: 10.3390/toxins8110334 (PMC5127130; doi:10.3390/toxins8110334)
Supplement: Supplementary file 1 [file toxins-08-00334-s001.pdf]

Supplementary Materials: Deoxynivalenol and Its Modified Forms: Are There Major Differences?

Arash Alizadeh, Saskia Braber, Peyman Akbari, Aletta Kraneveld, Johan Garssen and Johanna Fink-Gremmels

Comparison of Lactate dehydrogenase (LDH) leakage induced by DON, DON3G and DOM-1.

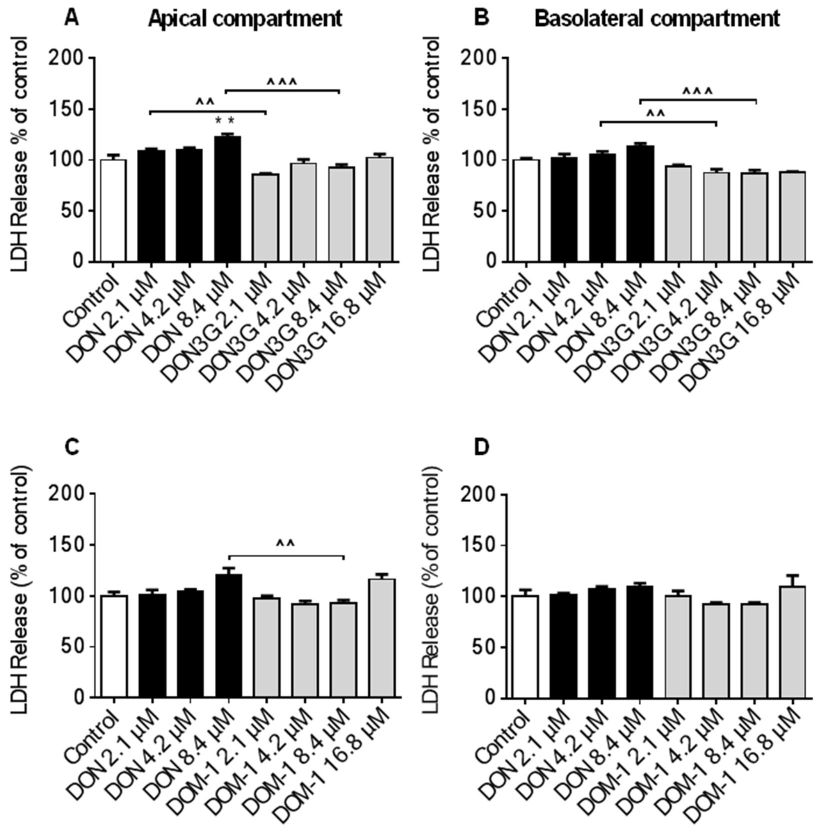

Figure S1. Cytotoxic effects of DON, DON3G and DOM-1 in Caco-2 cells. Differentiated Caco-2 cells on transwell inserts were exposed from the apical and basolateral compartment to increasing DON, DON3G (**A**,**B**) and DOM-1 **(C**,**D)** concentrations (2.1, 4.2, 8.4, 16.8 μM) for 24 h followed by evaluation of LDH release into the apical (**A**,**C**) and basolateral (**B**,**D**) compartment. Results are expressed as a percentage of LDH released by the control group as mean ± SEM. ** $p < 0.01$; significantly different from control group; ^^ $p < 0.01$, ^^^ $p < 0.001$ significantly different from corresponding DON group.
